# Ultrasound prenatal diagnosis of typical megacystis, microcolon, intestinal hypoperistalsis syndrome

**DOI:** 10.1002/ccr3.1481

**Published:** 2018-03-12

**Authors:** Natalia Buinoiu, Anca Panaitescu, Mihaela Demetrian, Sebastian Ionescu, Gheorghe Peltecu, Alina Veduta

**Affiliations:** ^1^ Filantropia Clinical Hospital Bucharest Romania; ^2^ MS Curie Children Hospital Bucharest Romania

**Keywords:** Intestinal hypoperistalsis, megacystis, microcolon, prenatal diagnosis

## Abstract

In the presence of megacystis in the second half of pregnancy, with increased amniotic fluid, especially in a female fetus, the most likely diagnostic result is megacystis, microcolon, intestinal hypoperistalsis syndrome, MMIHS. In these cases, the diagnosis of MMIHS should be strongly considered instead of lower urinary tract obstruction.

## Introduction

Megacystis, microcolon, intestinal hypoperistalsis syndrome is a rare congenital condition characterized by nonobstructed distended bladder, microcolon, and decreased intestinal peristalsis (intestinal dismotility). This condition can be suspected during pregnancy, based on ultrasound findings. After birth, the newborn presents with symptoms of intestinal obstruction and difficulties in bladder evacuation.

The syndrome was described by Berdon and colleagues in 1976 1, and a few hundred cases have been reported since then, in the pediatric literature 2, 3.

When diagnosed prenatally, MMIHS usually presents with enlarged fetal bladder (megacystis) and normal or increased amniotic fluid. Female gender is an important argument in favor of the diagnosis—the prevalence of this condition is three to four times higher in female fetuses than male fetuses 4.

## Case Report

A 28‐year‐old multipara was referred to our Fetal Medicine Department at 24 weeks of gestation, for fetal megacystis. During a routine ultrasound scan at 22 weeks, a large cystic mass had been noticed in the fetal pelvis; the initial diagnosis had been made for megacystis in a female fetus. The patient had delivered two healthy babies previously, and she had no history of consanguinity or any familial genetic disease.

**Figure 1:**
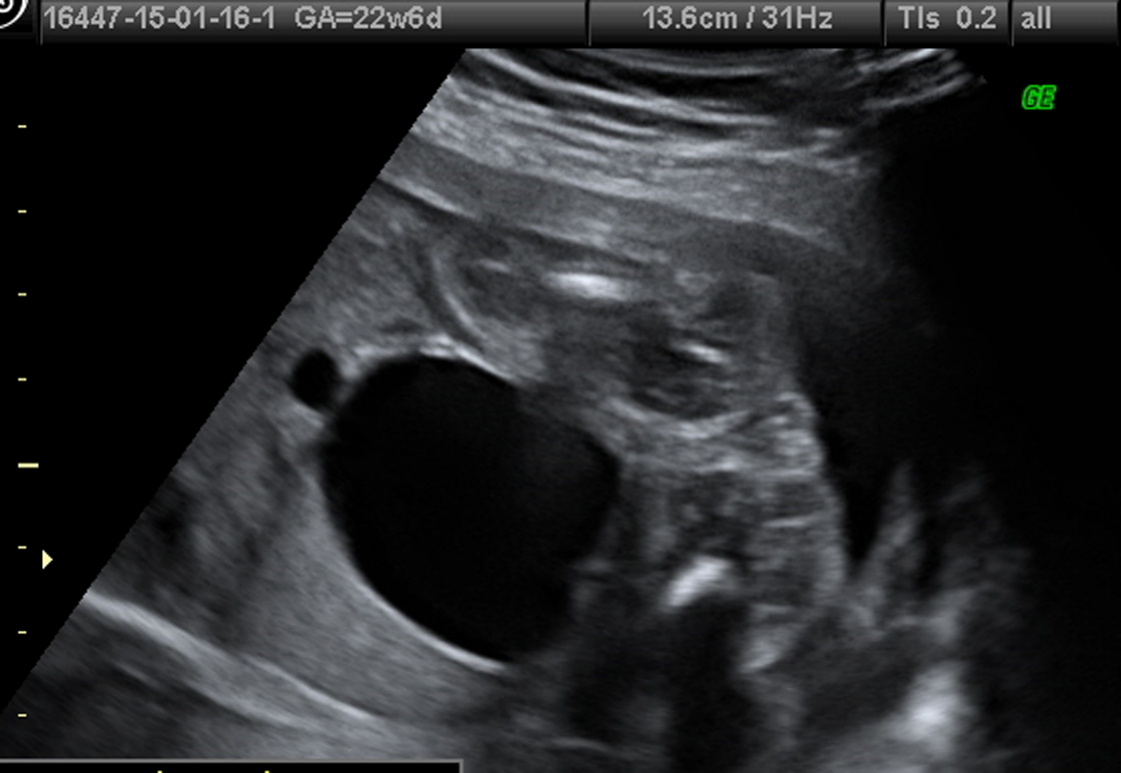
Coronal view of the pelvis and abdomen, showing megacystis in a female fetus, at 22 weeks of gestation.

We confirmed the megacystis (persistently enlarged fetal bladder with the maximum diameter of 5.1 cm) in an appropriately grown female fetus (Fig. 1). We also noticed mild bilateral hydronephrosis; the amniotic fluid was normal on our initial ultrasound examination. Fetal growth was on the 68th centile at presentation (the estimated fetal weight was 690 g).

**Figure 2:**
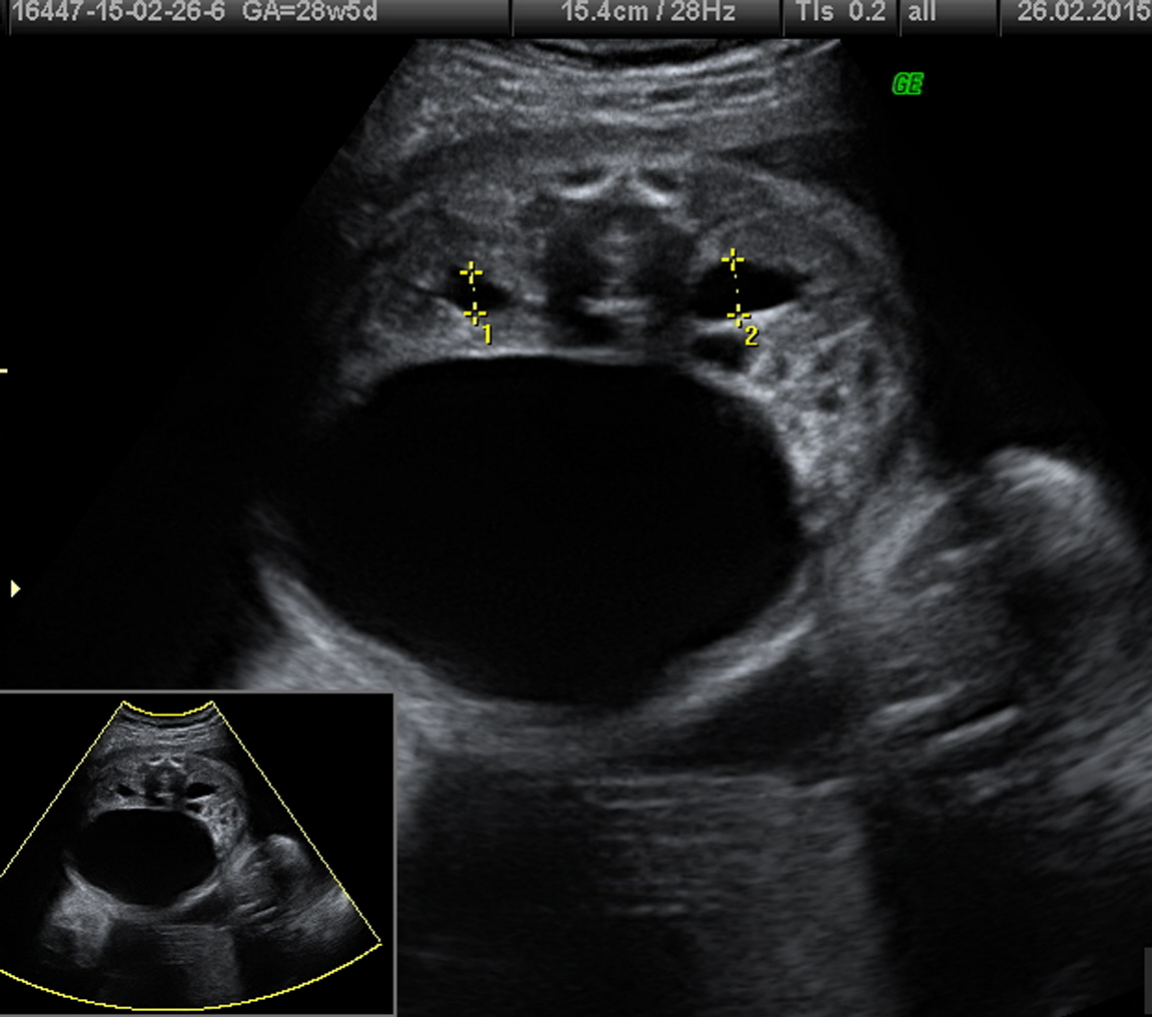
Transverse plane showing megacystis and mild hydronephrosis, at 28 weeks of gestation.

**Figure 3:**
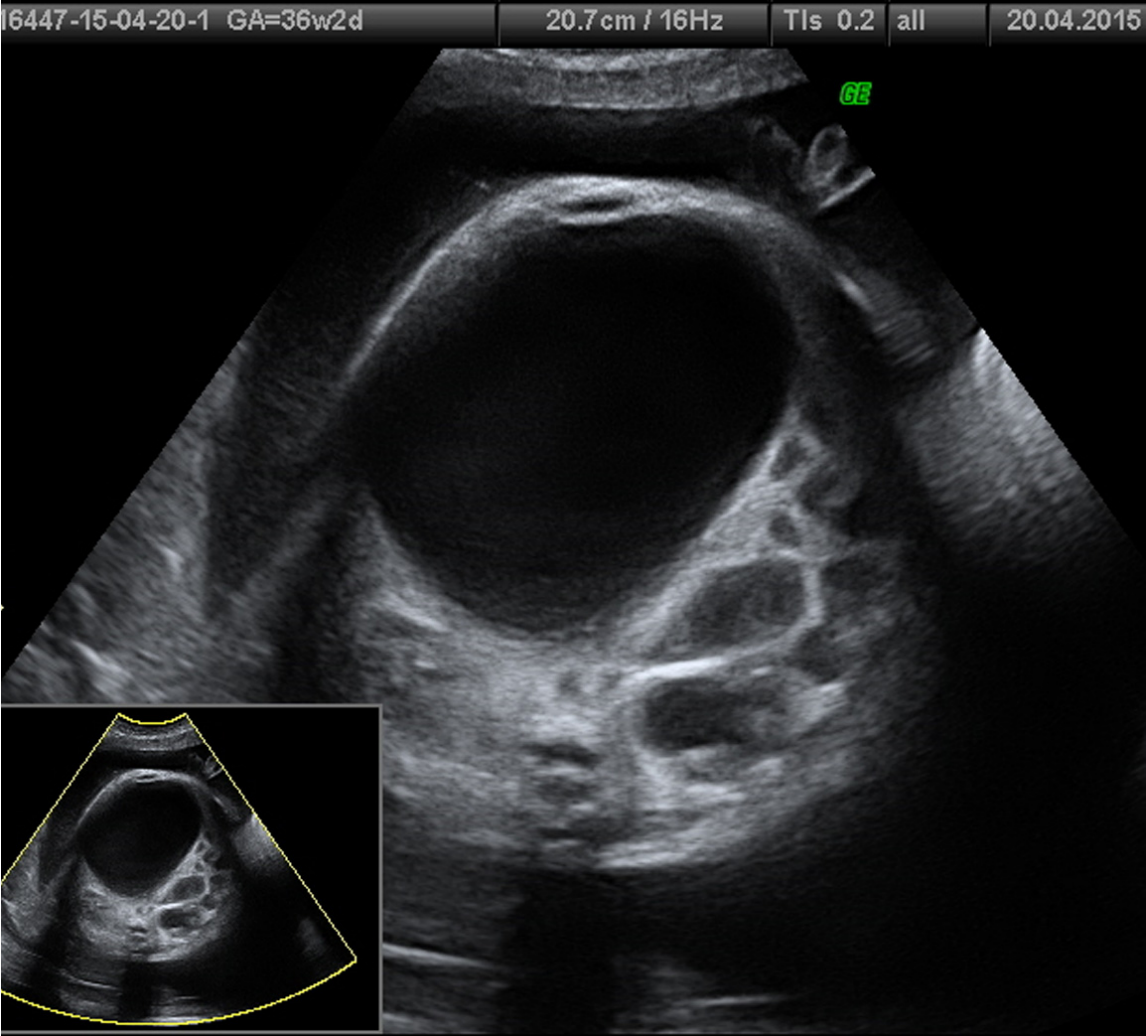
Transverse view of the fetal abdomen, showing megacystis and dilated bowel at 36 weeks of gestation.

The pathologies we considered at initial presentation were lower urinary tract obstruction (LUTO) and megacystis, microcolon, intestinal hypoperistalsis syndrome (MMIHS). The implications of the findings were discussed with the parents. We explained that, if the baby suffered of MMIHS, long‐term survival was unlikely. Amniocentesis for genetic testing was also discussed; we explained the indications and the limitations of prenatal genetic analysis, in this case. After counseling, the parents decided to continue the pregnancy and not to have genetic testing. Monthly follow‐up ultrasound scans were arranged. On subsequent scans, there was no progression of the hydronephrosis, while the amniotic fluid gradually increased (Fig. 2); polyhydramnios developed in the third trimester—the amniotic fluid index (AFI) was 32 at 36 weeks of gestation. The fetal bowel appeared dilated in the third trimester (Fig. 3). The evolution was therefore consistent with the diagnosis of megacystis, microcolon, intestinal hypoperistalsis syndrome.

Fetal growth and Doppler measurements were normal throughout the third trimester. As the patient had two previous cesarian deliveries, we planned delivery by cesarean section at term.

The membranes ruptured spontaneously at 38 weeks of gestation, and a 3650‐g fetus was delivered by cesarean section. An experienced neonatologist was present at birth.

**Figure 4:**
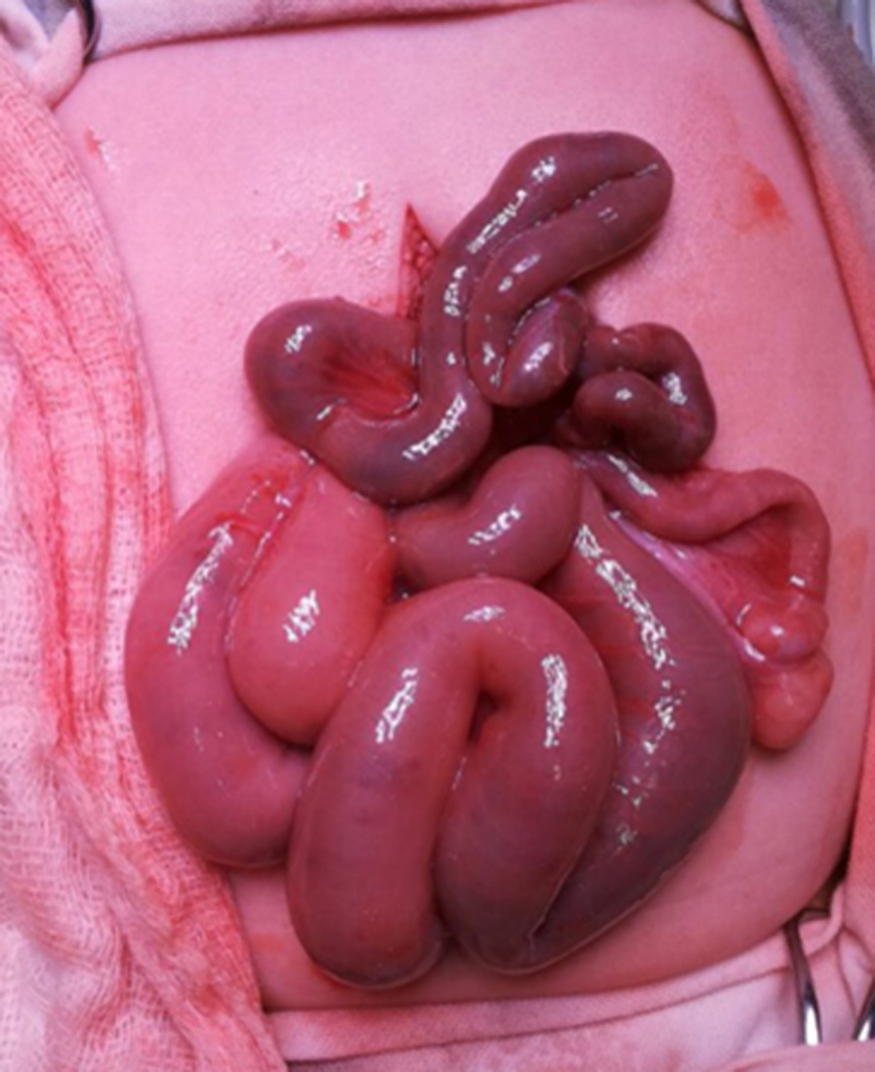
Laparotomy picture showing intestinal malrotation and microcolon, in the neonate.

MMIHS was confirmed postnatally. The baby girl was unable to pass meconium or to spontaneously void the bladder; the abdomen was distended by a hypogastric mass. Bladder catheterization temporarily resolved the abdominal mass. The baby was transferred to a pediatric surgery service. Surgical intervention (laparotomy) was decided at 3 days of age, because of unresolving intestinal obstruction. Intestinal malrotation and microcolon were seen on laparotomy (Fig. 4); an ileostomy was performed. Multiple full thickness biopsies of ileum and colon were performed during the surgical procedure; the histological findings were nonspecific.

On parenteral nutrition (TPN), the baby survived; she is now 30 months old. Her neurological development is normal, and she may be a candidate for intestinal transplantation.

## Discussion

This is a case of megacystis, microcolon, intestinal hypoperistalsis syndrome with typical prenatal presentation and with unusually favorable postnatal evolution.

MMIHS is a rare condition that associates a large bladder, a small colon, intestinal hypoperistalsis, and polyhydramnios; the condition predominantly affects female fetuses.

The literature on the prenatal diagnosis of MMIHS is not extensive. A recent review analyzed 50 cases of MMIHS, of which 26% were diagnosed prenatally; fetal megacystis was the most common initial ultrasound finding (88% of the prenatally diagnosed cases); increased amniotic fluid was frequently seen during the third trimester 5. The literature shows that the prenatal diagnosis of MMIHS remains challenging and that the data on the prenatal appearance of MMIHS are limited. Our case illustrates the evolution of a rare fetal disease.

The pediatric literature is more extensive and well analyzed. In 2016, Wymer and colleagues identified 135 reported cases 2, while in 2011, Puri and colleagues had identified 227 reported cases 3. A nationwide survey in Japan recently identified 28 cases of MMIHS 6. In 2013, Puri reviewed 47 cases of familial MMIHS 7. These reviews conclude that, while the prognosis of the pediatric patients with MMIHS is generally poor, recent advances in parenteral nutrition and transplant medicine significantly improved the survival of these patients.

Important insight has recently been gained into the genetics of the megacystis, microcolon, intestinal hypoperistalsis syndrome. MMIHS is considered part of the spectrum of visceral myopathy 8. De novo and familial cases have been documented, and both autosomal recessive and autosomal dominant inheritances have been proposed for MMIHS 9. Recently, genetic mutations responsible for the two types of MMIHS (autosomal dominant and autosomal recessive) have been identified. Autosomal dominant forms of MMIHS are caused by ACTG2 gene mutations 10, 11, 12, while autosomal recessive cases are caused by homozygous loss‐of‐function variants of MYH11 gene 13. Although many cases of MMIHS are associated with ACTG2 variants, not all the genes involved are yet known and mutations of other genes, besides MYH11, could cause autosomal recessive MMIHS 8. The mutations that are known to cause MMIHS can be identified by molecular analysis in affected individuals, but prenatal molecular studies are generally not useful in de novo cases.

The main clue for the prenatal detection of MMIHS is enlarged fetal bladder (megacystis). Fetal megacystis has an incidence of about 1:1500 4. It affects male fetuses predominantly, and it is most frequently caused by mechanical lower urinary tract obstruction (LUTO). LUTO is the first diagnosis to consider in cases with megacystis and oligohydramnios. However, in female fetuses with megacystis and polyhydramnios, MMIHS should be suspected, as LUTO rarely affects females and almost always causes oligohydramnios. MMIHS should not be ruled out in male fetuses with megacystis, especially if there is no oligohydramnios 14. Progressive hydronephrosis is usually suggestive of LUTO, but hydronephrosis can be seen in both MMIHS and LUTO 14. Fetal MRI studies can contribute to the prenatal diagnosis 14.

## Conclusion

We report a case of MMIHS that we diagnosed prenatally, in a female fetus with megacystis, dilated bowel, and increased amniotic fluid.

We think that, in the presence of megacystis in the second and the third trimester, with normal or increased amniotic fluid, in a female fetus, the most likely diagnostic result is megacystis, microcolon, intestinal hypoperistalsis syndrome, MMIHS. Quick prenatal diagnosis is therefore possible in these cases. Counseling should be conducted accordingly. LUTO, lower urinary tract obstruction, should be taken into consideration as differential diagnosis.

## Conflict of Interest

None declared.

## Authorship

NB, AP: contributed to the preparation and review of the manuscript, collected scientific data on the subject. NB: wrote the first version of the manuscript. MD, SI, GP, AV: managed the case and provided scientific data on the subject. AV: provided editing of the manuscript, wrote the final version of the article.
